# Inclusion of a GaAs detector model in the Photon Counting Toolkit software for the study of breast imaging systems

**DOI:** 10.1371/journal.pone.0270387

**Published:** 2023-06-08

**Authors:** Bahaa Ghammraoui, Katsuyuki Taguchi, Stephen J. Glick

**Affiliations:** 1 Division of Imaging, Diagnostics and Software Reliability, Office of Science and Engineering Laboratories, CDRH/FDA, Silver Spring, Maryland, United States of America; 2 Radiological Physics Division, The Russell H. Morgan Department of Radiology and Radiological Science, Johns Hopkins University School of Medicine, Baltimore, MD, United States of America; Accra Technical University, GHANA

## Abstract

We present an upgraded version of the Photon Counting Toolkit (PcTK), a freely available by request MATLAB tool for the simulation of semiconductor-based photon counting detectors (PCD), which has been extended and validated to account for gallium arsenide (GaAs)-based PCD(s). The modified PcTK version was validated by performing simulations and acquiring experimental data for three different cases. The LAMBDA 60 K module planar detector (X-Spectrum GmbH, Germany) based on the Medipix3 ASIC technology was used in all cases. This detector has a 500-*μ*m thick GaAs sensor and a 256 × 256-pixel array with 55 *μ*m pixel size. The first validation was a comparison between simulated and measured spectra from a ^109^Cd radionuclide source. In the second validation study, experimental measurements and simulations of mammography spectra were generated to observe the performance of the GaAs version of the PcTK with polychromatic radiation used in conventional x-ray imaging systems. The third validation study used single event analysis to validate the spatio-energetic model of the extended PcTK version. Overall, the software provided a good agreement between simulated and experimental data, validating the accuracy of the GaAs model. The software could be an attractive tool for accurate simulation of breast imaging modalities relying on photon counting detectors and therefore could assist in their characterization and optimization.

## Introduction

Clinical x-ray imaging equipment has been operating in energy integrating mode for more than a century. During the last few years, we have been on the verge of a paradigm shift in clinical x-ray detector technology, with future detectors enabling photon counting and energy discrimination. These Photon Counting Detector (PCD) systems have several advantages, particularly in breast imaging [1]. Previous studies have shown that photon counting imaging systems can reduce electronic noise and beam-hardening, improve contrast-to-noise ratio through energy weighting, improve radiation dose efficiency, and could enable high spatial resolution. Furthermore, simultaneous multi-energy data acquisition would allow differentiation among multiple contrast agents [2]. It is well known that most of the major computed tomography (CT) companies have been developing prototype PCD-CT systems recently, and last year the FDA cleared the world’s first PCD-CT scanner, the Siemens Naeotom Alpha. This current rapid development of PCD spectroscopic imaging systems is made possible by recent technological developments in integrated circuits for reading signals from solid state detectors, and advances in the capabilities of the maximum count rate, as well as the lower cost and improved reliability of larger semiconductor boards.

However, several technical challenges still must be overcome in order to realize the full potential of PCD. These include charge sharing, pulse pile-up, characteristic escape peaks, Compton scattering, and weighting potential cross talk, which can severely distort measured spectra and spatial resolution in such pixelated detectors [3, 4]. It is widely recognized that computer simulation codes and models can help, and they represent key components of the development, optimization and evaluation of such imaging systems. To date, only one available code has been developed and dedicated to modeling semiconductor-based PCDs. This code is part of the Photon Counting Toolkit (PcTK) software [5], which was developed mainly to model cadmium telluride (CdTe)-based PCDs. Other semiconductor-based x-ray PCD candidates, including silicon (Si) and gallium arsenide (GaAs), are under investigation and development and are not yet modeled in the PcTK. GaAs, specifically, is promising for breast imaging, since high quantum efficiencies are easily achievable within the mammography energy range (12 to 45 keV). Furthermore, its characteristic K-edges lie below the mammography-relevant energies (9, 10, and 12 keV), minimizing the likelihood of fluorescence x-rays escaping the pixels. However, its implementation in sizeable flat panel detectors has been limited by many challenges in fabricating a high-performance and uniform GaAs semiconductor. Nevertheless, recent technological developments in manufacturing high-purity GaAs crystal are noticed and several relatively larger 2D pixelated GaAs PCD have been developed. In addition, there is a lack of robust and comprehensive studies to optimize parameters for the GaAs detector for a breast imaging application.

In a recent study, Ghammraoui et al. showed that GaAs spectral mammography provides slightly improved or equivalent performance versus commercial mammography systems equipped with energy integrated detectors [6]. In that study, commercial mammography system exposure irradiation conditions were used, and no attempt was made to optimize parameters for the GaAs detector.

Such an optimization study could efficiently be conducted using a reliable experimentally validated GaAs PCD simulation. Appropriate optimization efforts could reduce development time and costs, and to our knowledge, no publicly available code is dedicated to simulating GaAs-based 2D pixelated detectors. In this work, we present an extension to the PcTK that enables realistic GaAs PCD simulation. We first describe the upgraded simulation parameters needed to model GaAs and then provide information on the three experimental procedures used for parameter optimization and code validation. Such code with GaAs simulation could promote PCD research in mammography, CT and digital breast tomosynthesis (DBT) modalities.

## Materials and methods

### Detector model

The PcTK code was extended to account for the simulation of a GaAs semiconductor with an atomic ratio As/Ga of 1. The extended version uses the same design concept used in PcTK version 3.2, described in detail in a previous study [5]; Here, we will give a brief description. The PcTK version 3.2 uses a cascaded parallel model to simulate how an x-ray incident on a pixel of interest deposited its energy in that pixel and its eight neighboring pixels through several physical phenomena that occurred throughout the detecting procedure. One x-ray photon may interact through photoelectric effect with or without fluorescence x-ray emission, or it may simply pass through the detector without interacting. As a result, one or two charge clouds can be produced at the interaction sites, which can be detected by multiple adjacent pixels. Neither Compton scattering nor Rayleigh scattering were included in the PcTK version 3.2. Consequently, one incident photon can create several counts when its energy is divided among many pixels, where the recorded energy at each pixel is lower than the original energy.

When the photon energy is split into more than one pixel, the recorded energy at each pixel is lower than the original energy, and one photon produces more than one count. This is called multiple counting. Consequently, PCD recorded data are spatially and energetically correlated. PcTK models record x-ray spectrum with such correlation. Users provide PcTK with several parameters that can vary according to detector specifications, such as detector thickness, pixel size, the size of the charge cloud *r*_0_, and the electronic noise *σ*_*e*_. Then, PcTK computes the probabilities of energy and counts recorded at 3x3 pixels when x-rays are incident onto somewhere within the central pixel and outputs a normalized covariance matrix for 1-keV-width energy windows or a given definition of energy window bins.

In this work, we kept each element identical to version 3.2 except for the inclusion of the GaAs interaction probabilities, with several assumptions described below, as it is meant for the energy range pertinent to breast imaging.

The GaAs model accounts for the probabilities of the different interaction phenomena in a GaAs sensor in different ways. We ignored Compton scattering interactions, since there is a low probability of these in the mammography energy range. Therefore, given an x-ray interaction absorbed within the detector, the conditional probabilities at energy E of photoelectric effect are assumed to be equal to 1. The conditional probability of K-shell void given photoelectric effect for both Ga and As was assumed to be 88% [7]. We also used experimentally measured conditional probability of fluorescence x-ray emission, given the K-shell void present (*W*_*k*_=0.528 for Ga and *W*_*k*_=0.589 for As) [8], and assumed fixed yields of 0.9 and 0.1 for K-alpha and K-beta x-ray fluorescence [9]. Finally, the mean travel distances of Ga and As fluorescence x-rays were assumed to be 42*μ*m and 16*μ*m, respectively [10]. Other parameters, such as detector thickness, pixel size *d*_0_, charge cloud radius *r*_0_ and the electronic noise parameter *σ*_*e*_ can vary according to detector specifications, and thus are to be input by the user.

## Validation

The modified PcTK version was validated by performing simulations and experimental data for three different cases. The LAMBDA 60 K module planar detector (X-Spectrum GmbH, Germany) based on the Medipix3 ASIC technology was modeled for simulations and used for validations using experimental measurements. This detector has a 500-*μ*m-thick GaAs sensor and a 256 × 256-pixel array with 55 *μ*m pixel size.

### Optimization study

**Fig 1:**
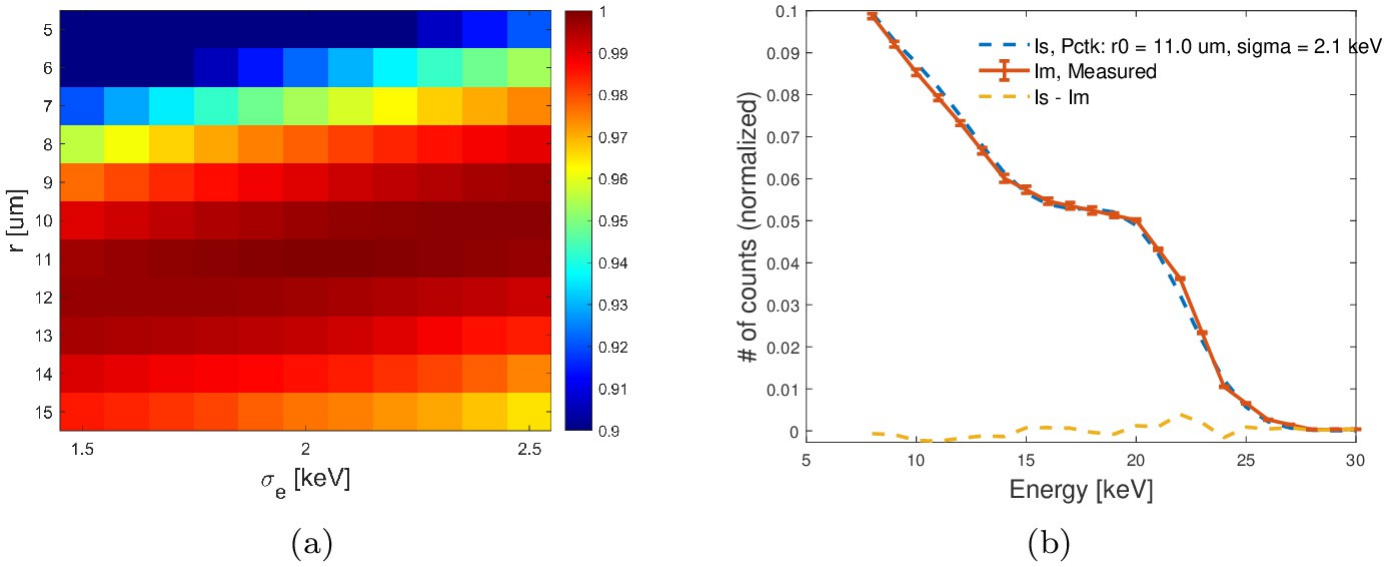
(a) shows the normalized cross correlation factors between the measured ^109^Cd spectrum and 100 simulated spectra with different *r*_0_ and *σ*_*e*_ model parameters. (b) comparison between the experimental and simulated spectra when using 11 *μ*m and 2.1 keV for *r*_0_ and *σ*_*e*_, respectively. The vertical error bars correspond to the ± standard deviation across the five measurements for each point.

The first experiment aimed for both validation and to obtain the optimum *r*_0_ and *σ*_*e*_ model parameters for simulating the LAMBDA 60 K module planar detector used in these studies. Experimentally, the spectrum emitted from a ^109^Cd sealed source was measured by sweeping the energy threshold from 5 to 30 keV with a decrement of 2 keV, and the sweeping process was repeated five times to acquire the mean spectra with lower variability. Detector pixel spectra were calculated by differentiation between successive images. The spectrum also was averaged over 400 randomly selected pixels. In addition, simulations were performed using different *r*_0_ and *σ*_*e*_ model parameters assuming monochromatic radiation of 22 keV (primary emission from ^109^Cd). The simulated *r*_0_ values ranged from 5 to 15 *μ*m with 1 *μ*m spacing; and *σ*_*e*_ values ranged from 0.5 to 2.5 keV with 0.1 keV spacing. The simulated detector pixel size and thickness were set to 55 *μ*m and 500-*μ*m, respectively. As a figure-of-merit, the normalized cross correlation factors between the measured and simulated spectra were used. The optimal parameter values that maximize the correlation factor were selected for the further measurements and were found to be 11 *μ*m and 2.1 keV for *r*_0_ and *σ*_*e*_, respectively as shown in Fig 1(a). It can be seen in Fig 1(b) that the experimental spectrum was correctly reproduced in the simulation by the updated tool using these optimum parameter values for *r*_0_ and *σ*_*e*_.

### Experimental validation using polychromatic x-ray spectra

**Fig 2:**
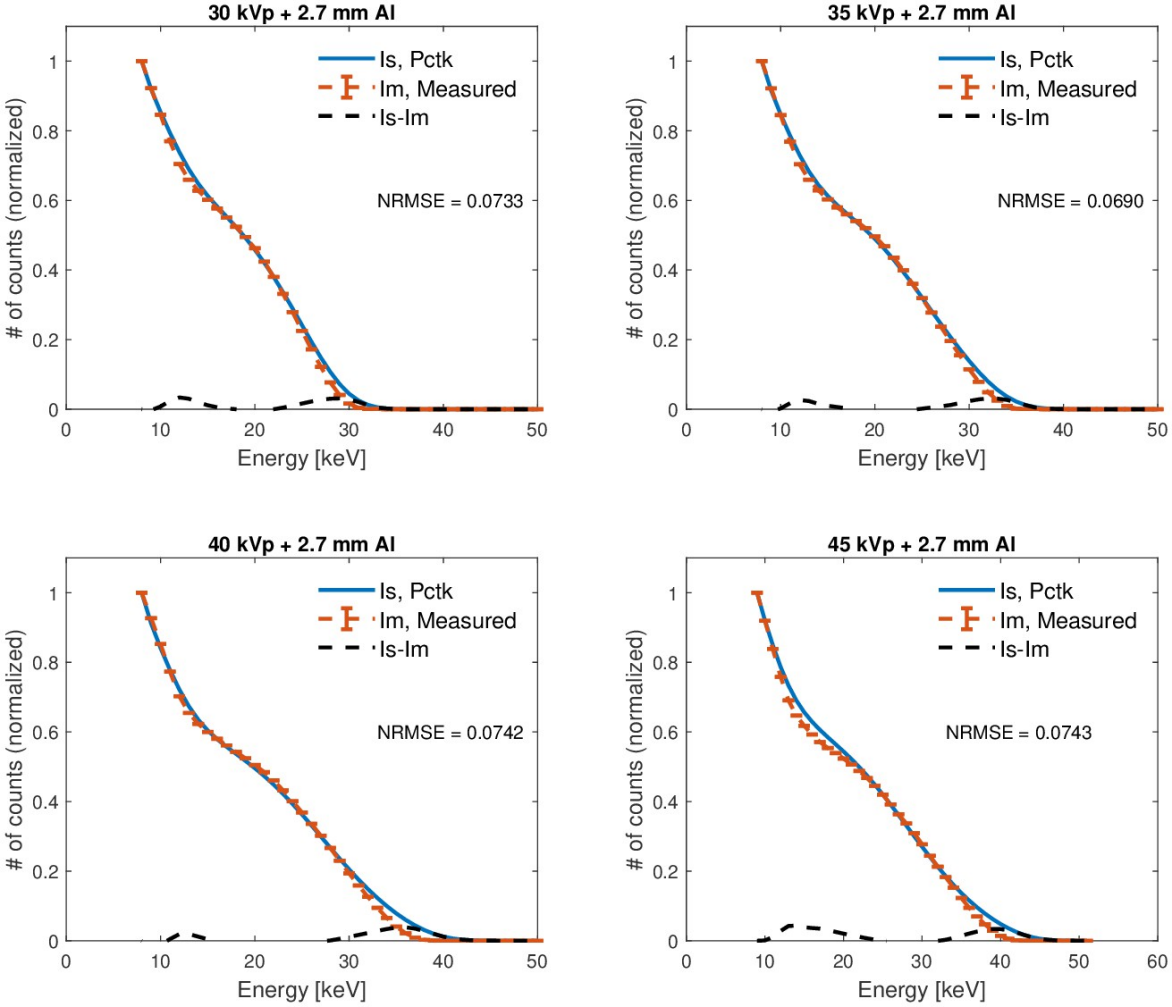
Comparisons between simulated and measured spectra with incident tungsten target X-ray spectra. The NRMSE between the two spectra is also reported. The vertical error bars correspond to the ± standard deviation across the five measurements for each point.

For validation in the second experiment, experimental and simulated mammography spectra were used to observe the performance of the GaAs version of the PcTK with polychromatic radiation used in conventional x-ray imaging systems. Polychromatic spectra emitted from an industrial X-ray tube with a tungsten target (Comet MXR-160/22) which operated at 30, 35 and 40 kVp, and 2 mA were measured and compared to simulated spectra using the GaAs version of the PcTK. The detector was placed 1 m away from the source, and 2.7 mm of aluminum filtration was placed in front of the source to mimic the mammography x-ray spectra transmitted through a breast. The spectra were measured by sweeping the energy threshold from 5 to 49 keV with a decrement of 2 keV, and the sweeping process was repeated several times to acquire the average and obtain better statistics. Final spectra were calculated by differentiation between successive images. The same spectra were also measured using the XR100T-CdTe Amptek (Amptek, Bedford, MA, USA) detector and corrected for spectral distortion using the simulated system response matrix described by Ghammraoui et al. [11]. The corrected spectra from the Amptek detector were the inputs to the GaAs version of the PcTK. As mentioned earlier, the optimal parameter values of *r*_0_ and *σ*_*e*_ found in the first study were used in the simulation. Fig 2 shows the simulated spectra using the PcTK and the measured spectra using the GaAs LAMBDA detector. Each spectrum was normalized to its maximum intensity value. The normalized root mean square error (NRMSE) were used to characterize the agreement between the measured (*I*_*m*_) and simulated (*I*_*s*_) spectra.

$$\begin{array}{c} NRMSE=\sqrt{\frac{1}{n}\sum_{i}{\left(\frac{I_{m}\left(E_{i}\right)-I_{s}\left(E_{i}\right)}{I_{s}\left(E_{i}\right)}\right)}^{2}}, \end{array}$$ (1)

The plots confirm good performance of the algorithm with polychromatic radiation. However, a slightly worse performance was observed for 40 and 45 kVp spectra, especially at the higher energy range. The origin of this small disagreement can be explained by different factors, including inaccurate inputs in the incident spectra, which were estimated from the Amptek one pixel detector measurements followed by the spectral analysis correction, inhomogeneity of the spectral response in different pixels, and inaccurate inputs for *r*_0_ and *σ*_*e*_, and adding Compton to the detector model might improve the performance at high energy.

### Experimental validation of the spatio-energetic model using single events analysis

**Fig 3:**
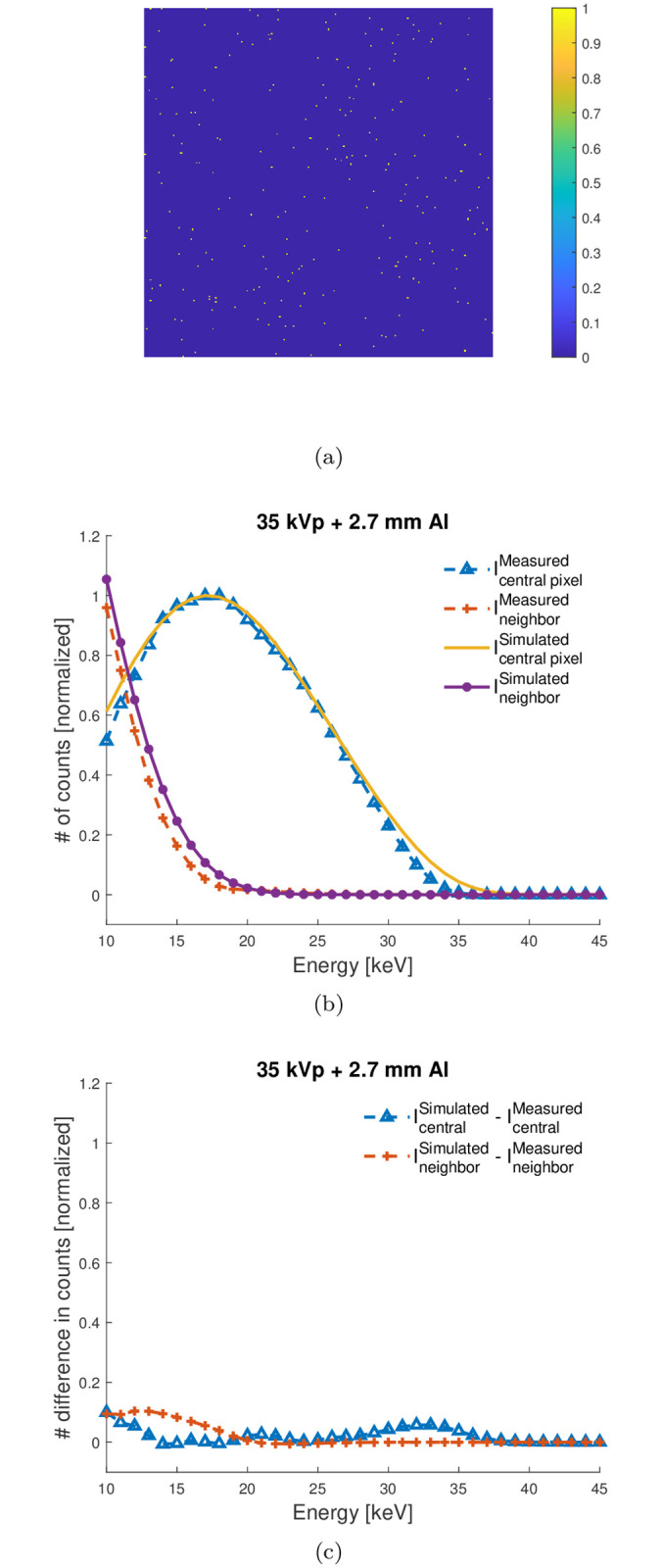
(a) Sample image of single events interactions recorded at energy threshold of 15 keV. (b) and (c) Comparison between the experimental and simulated spectra estimated using the single events analysis method when using 11 *μ*m and 2.1 keV for *r*_0_ and *σ*_*e*_, respectively.

The third experiment involved validating the spatio-energetic model of the GaAs version of the PcTK using single events analysis [12]. Single events analysis was performed by exposing the detector to low fluence in order to generate point-like images where only clusters from one absorbed x-ray were present, as shown in Fig 3(a). This was done by placing the detector 7 m away from the source and reducing the x-ray generator-operated current and voltage to 0.1 mA and 35 kVp, respectively. For each energy threshold acquisition, 1,800 frames were recorded, and the frame time was set to 1 ms. The analysis was performed on each frame separately to account for the number of events in which: (1) *N*_*single*_(*E*_*threshold*_) we had only one separate pixel activated and (2) *N*_*multiple*_ we had multiple adjacent pixels activated. The single events experiment was simulated using a 3×3 pixels detector and only irradiating the central pixel. Such analysis can be used to estimate the spectra recorded by the central pixel (irradiated), *I*_*central*_(*E*_*threshold*_) and its neighboring pixels *I*_*neighbor*_(*E*_*threshold*_) separately.

Experimentally, *I*_*central*_(*E*) and *I*_*neighbor*_(*E*) can also be estimated from the measured *N*_*single*_(*E*) and *N*_*multiple*_(*E*) values, using the following equations:

$$\begin{array}{c} I_{central}\left(E_{threshold}\right)\approx N_{single}\left(E_{threshold}\right)+N_{multiple}\left(E_{threshold}\right) \end{array}$$ (2)

$$\begin{array}{c} I_{neighbor}\left(E_{threshold}\right)\approx N_{multiple}\left(E_{threshold}\right) \end{array}$$ (3)

Fig 3 shows a comparison between the estimated and simulated *I*_*central*_(*E*) and *I*_*neighbor*_(*E*) spectra (≈ differentiation between two successive *I*_*central*_(*E*_*threshold*_) and *I*_*neighbor*_(*E*_*threshold*_), respectively). In this work, each *I*_*central*_(*E*) spectrum was normalized to its maximum value and the same normalization factor was then used to normalize its corresponding *I*_*neighbor*_(*E*).

As can be observed, the measured spectra are correctly reproduced using the GaAs version of the PcTK. Although some disagreements could be observed, especially at the higher ends of the spectra, the plots confirm good overall performance of the model used, and these disagreements can be considered negligible. Better performance could be possibly achieved by tuning the *r*_0_ and *σ*_*e*_ model parameters and adding Compton to the detector model.

There are some limitations with this study. Fig 2 shows a comparison between the simulated and measured energy spectra obtained with the tungsten target x-ray spectra. Each of these spectra were normalized to their maximum intensity value. In doing this normalization, we aim to solely compare spectra shape of the measured and simulated data. Ideally, we could have divided each of the measured and simulated spectra by the incident (on the detector face) spectra, giving the detection efficiency as a function of energy (which is not what we report in Fig 2). Determining the incident fluence as a function of energy is a challenging measurement which requires accurately estimating a number of parameters including source-to-detector distance, solid angle from the source subtended by the detector pixel, small rotations of the azimuthal or co-polar angles of the imaging geometry, and accurate activity measurement of the Cd-109 source with a well counter detector. From our experience, all of these estimations add a small to moderate variability to the measurement making absolute comparison of experimental to simulated detection efficiency difficult without the introduction of moderate size error in the result. In the study described herein, we use a 3×3 pixel region to model interpixel interactions. We rationalize this decision by noting that for detectors with pixel size of 55 *μ*m, we estimate that approximately only 5-10% of Ga K-edge x-rays (42 *μ*m mean free path) emitted from the center pixel will produce K-edge characteristic x-rays of Ga that travel outside of the 3×3 pixel region. Of course, this probability would be larger for detectors with smaller pixel size, so caution must be taken if using this model for detectors with smaller pixel size. Nonetheless, all commercial mammography systems use detectors with pixel size larger than 55 *μ*m.

## Conclusion

In this study, we presented an extension to the PcTK, dedicated to the simulation of a realistic GaAs PCD. The extended version was evaluated by comparison between simulated and measured spectra. The first validation was by comparison between simulated and measured EDXRD spectra from a ^109^Cd radionuclide source and was also aimed at obtaining the optimum model parameters for simulating the LAMBDA 60 K module planar detector used in this study. The second validation involved evaluating the performance of the GaAs version of the PcTK with polychromatic radiation used in conventional x-ray imaging systems. The third validation experiment was to validate the spatio-energetic model of the GaAs version of the PcTK using single events analysis. Overall, the software provided a good agreement between simulated and experimental data, validating the accuracy of the GaAs model. The PcTK can now be used to design, study and optimize the parameters affecting the image quality of breast CT, DBT and mammography imaging systems with GaAs-based photon counting detectors.

## Disclosures

The mention of commercial products, their sources, or their use in connection with material reported herein is not to be construed as either an actual or implied endorsement of such products by the Department of Health and Human Services.
